# Global respiratory tumor mortality correlation study with economic level, 2000–2019

**DOI:** 10.3389/fpubh.2025.1647634

**Published:** 2025-08-19

**Authors:** Mingjie Wang, Zhiyuan Chen

**Affiliations:** ^1^Department of Emergency Medicine, Qilu Hospital of Shandong University, Jinan, China; ^2^Shandong Provincial Clinical Research Center for Emergency and Critical Care Medicine, NMPA Key Laboratory for Clinical Research and Evaluation of Innovative Drug, Medical and Pharmaceutical Basic Research Innovation Center of Emergency and Critical Care Medicine, China’s Ministry of Education, Shandong Provincial Engineering Laboratory for Emergency and Critical Care Medicine, Key Laboratory of Cardiopulmonary-Cerebral Resuscitation Research of Shandong Province, Qilu Hospital of Shandong University, Jinan, China; ^3^Department of Infection Control, Provincial Hospital of Shandong First Medical University, Jinan, Shandong, China

**Keywords:** respiratory tumor, mortality rate, economic, gender difference, formulation

## Abstract

**Objective:**

The study aimed to investigate the relationship between global tracheobronchial lung cancer mortality rates and economic levels and assess the associated regional economic burden. Understanding these associations is crucial for global health resource allocation, informing cancer prevention and control strategies, and providing data to support the development of lung cancer and economic policies worldwide.

**Methods:**

We analyzed respiratory cancer mortality data (International Classification of Diseases (ICD)-10 codes C33-C34) obtained from the World Health Organization (WHO) Mortality Database (2000–2019). Age-standardized mortality rates (ASMRs) were calculated to describe the spatiotemporal distribution characteristics. Non-parametric comparisons (Wilcoxon rank-sum test) were performed to assess gender differences in mortality. Spearman’s partial rank correlation analysis was performed to evaluate the association between national income levels (World Bank classification) and disease mortality.

**Results:**

The core cohort included 50 countries with sufficient data. The global mean ASMR for respiratory cancers showed a decreasing trend from 2000 to 2019. Countries included in the Global Respiratory Tumor Mortality Registry System (GRTMRS) were predominantly high-income (68%) and European (52%). A significant positive correlation was observed between income levels and respiratory cancer mortality (Spearman’s *ρ* = 0.422, *p <* 0.001). ASMRs were consistently and significantly higher among male individuals than female individuals (Wilcoxon rank-sum test, *p* < 0.001).

**Conclusion:**

Between 2000 and 2019, global tracheobronchial lung cancer mortality appeared to be positively correlated with national economic level, particularly in high- and middle-income countries. Age-standardized mortality rates were significantly higher in male individuals than in female individuals. Paradoxically, these findings suggest that increasing economic development may be associated with elevated respiratory cancer mortality rates, emphasizing the critical need for balanced prevention strategies tailored to both high- and low-income settings.

## 1 Introduction

In 2020, respiratory cancers accounted for an estimated 2.6 million new cases (13.5% of all cancer cases) and 2 million deaths (20.2% of cancer-related mortality) globally (1). These malignancies can develop throughout the respiratory tract, affecting areas from the upper regions (nasal cavity and pharynx) to the lower regions (trachea, bronchi, and lungs), and primarily include laryngeal, tracheal, bronchial, and lung cancers. Tracheal, bronchial, and lung (TBL) cancers constitute the predominant subtype (2). Lung cancer, in particular, is the most commonly diagnosed cancer globally, with approximately 2.5 million new cases annually. Significantly, 2020 estimates indicate stark gender disparities in lung cancer mortality, with approximately 1.8 million deaths among men and 596,000 among women (3). This pronounced gender difference underscores the necessity for targeted prevention and control strategies that address the sex-specific disease burden. Environmental and occupational hazards linked to industrialization, such as ambient air pollution (e.g., PM2.5) and exposure to carcinogens such as asbestos and heavy metals, directly elevate the risk of cancer (4). Concurrently, an inverted U-shaped curve in tobacco consumption—characterized by rising smoking prevalence during economic transitions in low- and middle-income countries (LMICs)—further escalates the burden of respiratory cancer. Compounding these risks, the inequitable distribution of healthcare resources creates divergent challenges: high-income countries (HICs) may experience over-medication despite having access to early diagnostics such as low-dose computed tomography (LDCT) screening (5), while LMICs face preventable deaths due to delayed diagnosis and limited access to treatment.

In addition to their profound impact on human health, respiratory cancers impose substantial economic burdens due to their chronic nature, lengthy treatment regimens, and high costs (6). For instance, lung cancer, classified broadly as non-small cell lung cancer (NSCLC) (80–90% of cases) and small cell lung cancer (SCLC), incurs significant direct costs in advanced stages (7). Management costs for advanced NSCLC arise primarily from hospitalization, pharmaceuticals, and managing treatment-related adverse events (AEs). The introduction of advanced therapies (e.g., targeted agents, immunotherapies) has markedly increased direct costs per patient over the past decade. European studies report that these costs increased from approximately €16,000 in 2015 to €58,974 in 2023 (8).

Despite this context, analyses of the global distribution of respiratory cancer mortality in relation to economic levels, particularly concerning gender and regional income disparities, remain limited. There is a notable paucity of research examining the association between respiratory cancer mortality and economic status across different world regions. Therefore, in this study, we used mortality data for respiratory tumors from 2000 to 2019, obtained from the World Health Organization (WHO) Mortality database, alongside global economic income levels, to analyze the correlation between global income levels and respiratory tumor mortality rates, as well as sex differences. This study provides a reference basis for the prevention and control of respiratory tumors worldwide.

## 2 Materials and methods

### 2.1 Data sources

This study extracted respiratory tumor mortality data from 2000 to 2019 from the WHO Mortality Database, which compiles cause-of-death statistics by country/territory, year, sex, and age.^1^ The Global Economic Income Data Classification categorizes countries, regions, and territories based on the Atlas per capita gross national income (GNI) estimates for 2019 (Data source: World Bank list of economies released in June 2020.) Between 2000 and 2019, disease and cause-of-death data were harmonized across countries using the International Classification of Diseases (ICD)-9 and ICD-10 coding systems. Meanwhile, the age-standardized death rate (ASDR) was calculated using the world standard population structure provided by the World Health Organization (WHO) to eliminate the influence of differences in the age distribution of the population on the results and to significantly improve the comparability of the data across geographical areas and time periods. This study was limited by the nature of the WHO data source, which included only countries and regions with ≥65% civil registration completeness. Therefore, it failed to include a sample of low-income countries, and the results could not be extrapolated to these regions.

### 2.2 Data analysis and methods

This study describes the characteristics of the spatial and temporal distribution of age-specific mortality rates for respiratory tumors, with these mortality rates described as a central trend at the median (interquartile spacing).

To reveal the gender difference characteristics in mortality rates, non-parametric intergroup comparisons of respiratory tumor mortality rates between male and female groups were performed using the Wilcoxon rank-sum test based on the regional divisions and country groupings established by the World Health Organization (WHO).

To identify the complex relationship between income levels and disease mortality, partial Spearman rank correlation analysis was performed. Potential inflection points of mortality rates over time were identified by constructing a piecewise regression model (PRM), and the change in slope and the difference in slope before and after the inflection point were calculated separately to assess the stage-specific characteristics of trend evolution.

### 2.3 Statistical analysis

All data were statistically analyzed using SAS statistical software (version 9.4), and count data were expressed as the number of cases or percentages, which were statistically analyzed using the χ2 test, with a test level of *α* = 0.05 (two-sided).

## 3 Results

### 3.1 Data screening

**Figure 1:**
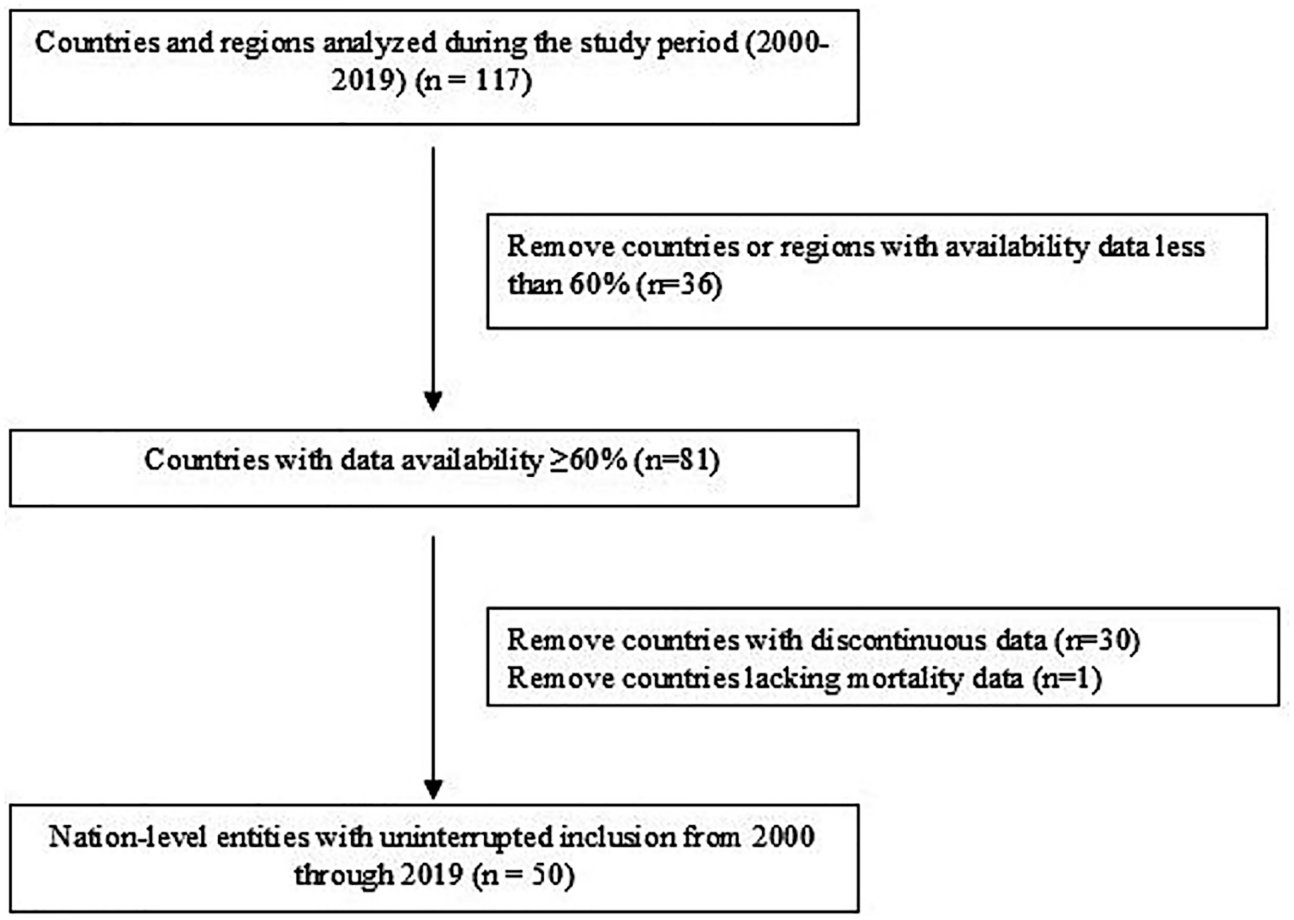
Flow diagram of the data search process.

This study utilized global surveillance data on morbidity and mortality related to tracheal, bronchial, and lung (TBL) cancers (ICD-10: C33-C34) from 2000 to 2019. The initial dataset comprised 117 countries and territories. Data selection was conducted through a two-stage screening process: (1) Initial Screening (Data Completeness): Countries with data availability for less than 60% of the study period (2000–2019) were excluded. This resulted in the retention of 81 countries, which formed the initial analysis set (see Supplementary Table 1 for details on the starting year, data completeness cycle, and years with missing data for each included country). (2) Continuous Reporting Validation: Countries retained after Stage 1 were further required to have continuous mortality data reported throughout the entire 20-year period (2000–2019). A total of 30 countries that did not meet this criterion were excluded. 3. Critical Data Verification: The final cohort was assessed for the presence of essential variables. Dominica was excluded due to missing data on the age-standardized mortality rate (ASMR) per 100,000 standard population. Following this screening process, 50 countries were included in the core analytical cohort (see Supplementary Table 1 and Figure 1).

### 3.2 Global trends in standardized mortality rates for respiratory tumors

**Figure 2:**
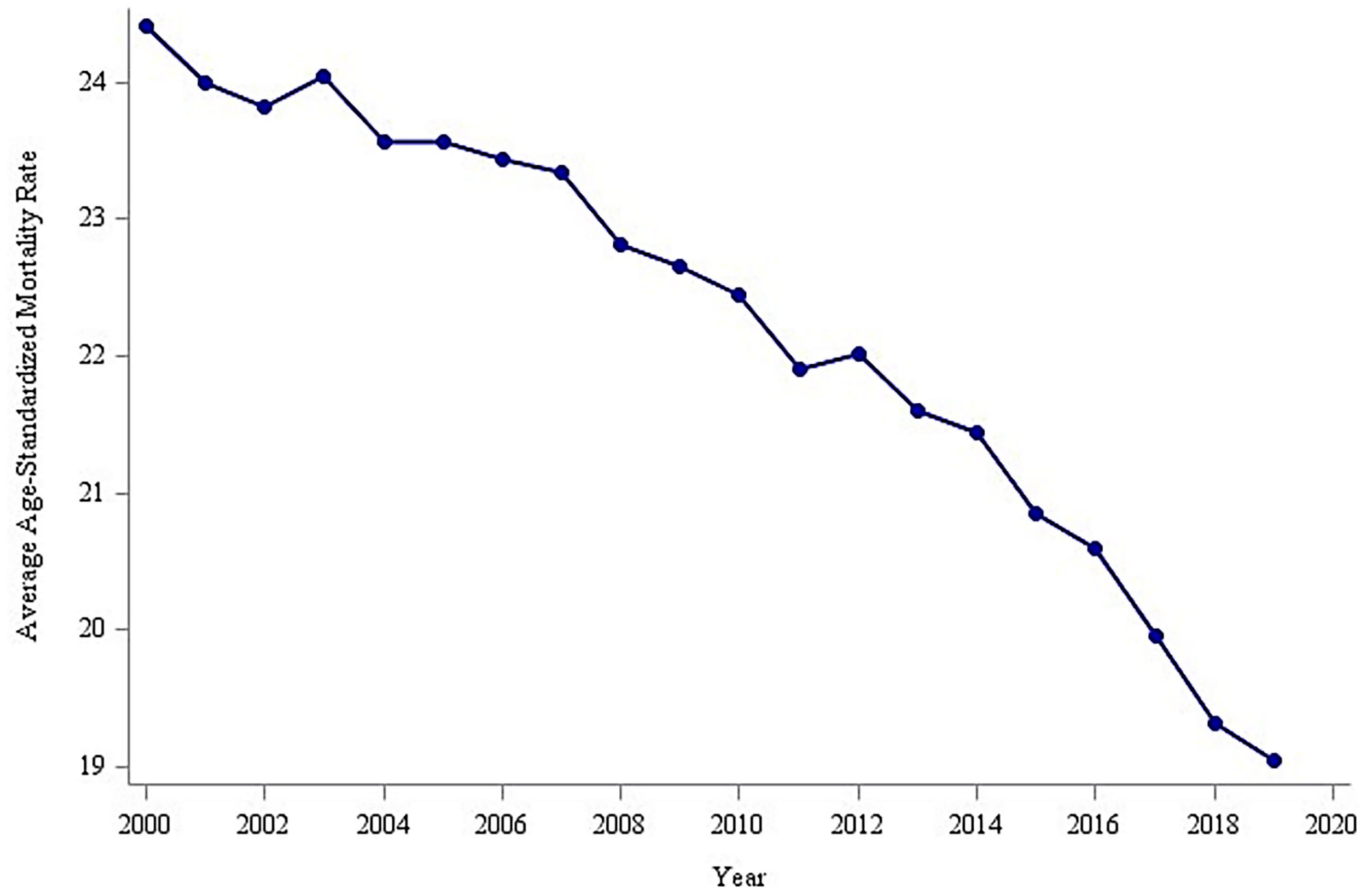
Global trends in age-standardized mortality rates for respiratory tumors.

**Figure 3:**
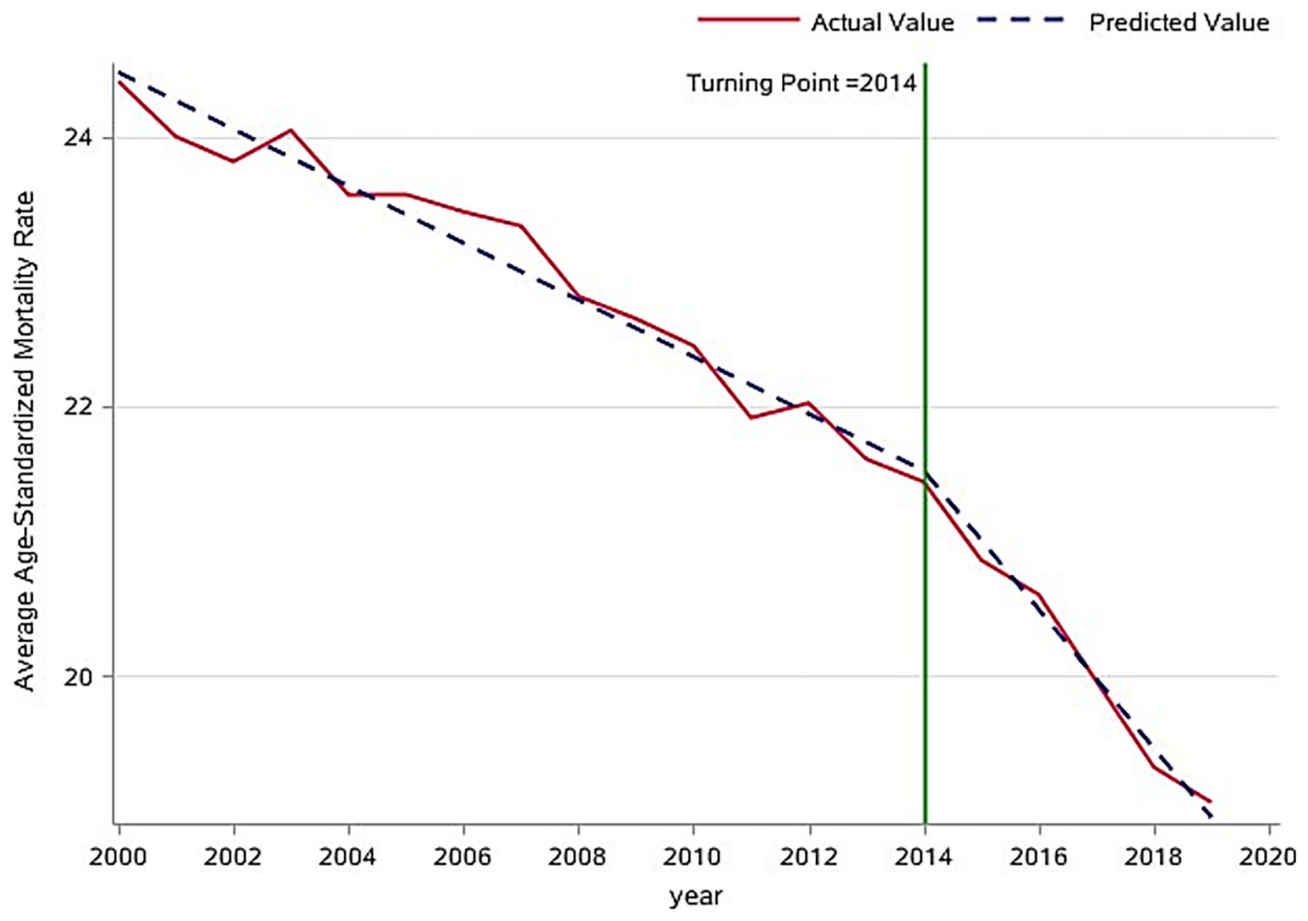
Inflection point analysis of global trends in age-standardized mortality rates for respiratory tumors.

The analysis of overall trends revealed a significant decline in the global average age-standardized mortality rate (ASMR) for tracheal, bronchial, and lung (TBL) cancers between 2000 and 2019, decreasing from 24.0 to 19.0 per 100,000 population (Figure 2). To identify potential shifts in this trend, a piecewise regression model was employed, with the inflection point year determined by minimizing the residual sum of squares. This analysis suggested 2014 as a potential inflection point. However, a subsequent F-test indicated that this inflection point was not statistically significant (*p* > 0.05). Despite this, the model segmented the time series into pre-inflection (2000–2014) and post-inflection (2015–2019) periods for further analysis of trend changes. Estimated average annual rates of change in ASMR were calculated for each segment (Figure 3). Before 2014, the ASMR declined significantly at an average rate of −0.211 per 100,000 population per year (*p* < 0.05). Following 2014, the rate of decline accelerated significantly to −0.515 per 100,000 population per year (*p* < 0.05). A formal test of the difference in slopes confirmed that the post-2014 decline was significantly faster than the pre-2014 decline (difference = −0.304 per 100,000 population per year; *p* < 0.05) (Table 1). This acceleration suggests that factors emerging or intensifying around 2014 may have coincided with the accelerated improvement in TBL cancer mortality trends globally, particularly in high- and middle-income countries.

**Table 1:** Parameter estimates from the segmented regression models of age-standardized mortality rates for global respiratory tumors (inflection point year = 2014).

Parameter	Estimate	Standard error	T	*p*
Intercept	21.521	0.079	272.53	<0.0001
Pre-inflection slope	−0.211	0.010	−21.37	<0.0001
Post-inflection slope	−0.515	0.032	−16.00	<0.0001
Slope difference	−0.304	0.039	−7.88	<0.0001

### 3.3 Characteristics of cross-regional respiratory tumor mortality and economic income distribution

The analysis of the 50-country core cohort revealed significant geographic and economic disparities in representation within the Global Respiratory Tumor Mortality Registry System (GRTMRS). High-income countries (68%, *n* = 34) and European nations (52%, *n* = 26) were substantially over-represented (Table 2 and Supplementary Table 2), indicating that the GRTMRS primarily reflects high-resource healthcare settings. This inherent bias was likely exacerbated by the exclusion of low-income countries due to data accessibility limitations.

**Table 2:** Distribution characteristics of the study cohort (*n* = 50).

Classification	Category	Number of Countries	Proportion (%)
Income level	High-income	34	68.0
	Upper-middle-income	14	28.0
	Low- and middle-income	2	4.0
	Low-income	0	0.0
Geographical region	Africa	1	2.0
	Asia	8	16.0
	Central and South America	10	20.0
	Europe	26	52.0
	North America and the Caribbean	5	10.0

Country income classifications are based on the World Bank’s 2020 criteria; geographic areas are based on the WHO Global Health Observatory (GHO) classifications.

**Figure 4:**
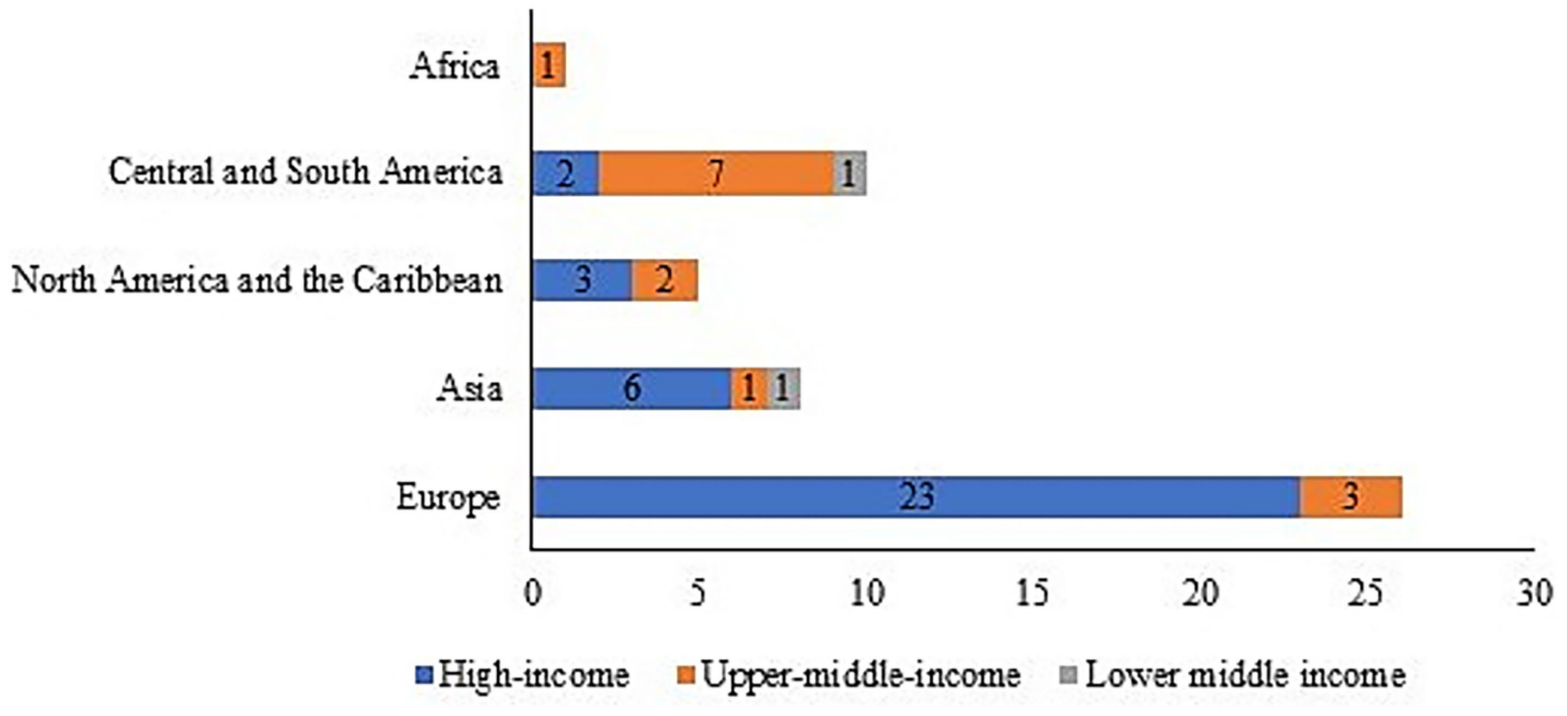
Distribution of the Included countries by income level and geographical region (*n* = 50).

Regional stratification further highlighted these imbalances (Figure 4): High-income countries (*n* = 34): The majority of these countries are situated in Europe (67.6%, 23/34) and Asia (17.6%, 6/34); upper-middle-income countries (*n* = 14): These countries are primarily clustered in Latin America and the Caribbean (50.0%, 7/14) and Europe (21.4%, 3/14); and LMICs (*n* = 2, 3.9%): This category includes one country each from Asia and Latin America/the Caribbean—both of which are regions facing a considerable burden of respiratory tumors. This minimal inclusion underscores the significant underrepresentation of LMICs. Notably, only one upper-middle-income African country was included (7.1% of upper-middle-income cohort, *n* = 1/14), highlighting critical gaps in mortality surveillance continuity across sub-Saharan Africa. These data establish a baseline for analyzing cross-regional differences in respiratory tumor mortality while explicitly acknowledging the cohort’s limitations regarding LMIC and African representation.

### 3.4 Correlation analysis between income and respiratory tumor mortality across regions

A partial Spearman rank correlation analysis was performed to assess the association between national income levels and respiratory cancer mortality across regions. As shown in Table 3, income status exhibited a significant positive correlation with respiratory cancer mortality, specifically within upper-middle-income countries (Spearman’s *ρ* = 0.422, *p* < 0.001). This positive association remained statistically significant after adjusting for country and sex (Partial Spearman’s ρ = 0.237, *p* < 0.001). These findings suggest a potential positive relationship between income levels and respiratory cancer mortality in middle- and high-income country regions globally. Stratifying countries by age-standardized mortality rate (ASMR) revealed that those with high mortality rates (ASMR ≥ 30.0 per 100,000 population) were predominantly high-income countries (70% of this group) and were within in Europe (70% of this group) (see Supplementary Table 3 and Supplementary Figure 1 for visualization).

**Table 3:** Sex differences in tracheal, bronchial, and lung cancer mortality rates by region and income group (2000–2019, Wilcoxon rank-sum test).

Classification	Category	Total	Male	Female	*p*-value
Income level	High-income	25.41 (20.33,29.76)	38.92 (30.32,49.87)	12.76 (9.28,17.89)	<0.0001
	Upper-middle-income	11.06 (8.05,28.5)	17.1 (11.26,45.34)	6.21 (4.51,11.04)	<0.0001
	Low- and middle-income	8.23 (4.85,12.38)	13.09 (6,22.16)	4.49 (3.9,4.85)	<0.0001
Geographical region	Africa	10.89 (10.21,11.45)	18.46 (16.29,19.69)	5.5 (5.03,5.78)	<0.0001
	Asia	19.43 (13.46,23.68)	30.81 (22.94,38.85)	9.54 (5.59,12.1)	<0.0001
	Central and South America	9.53 (6.15,12.62)	13.79 (8.33,18.07)	5.08 (4.16,8.21)	<0.0001
	Europe	27.36 (23.84,31.96)	46.57 (36.25,55.33)	14.04 (10.15,17.68)	<0.0001
	North America and the Caribbean	28.45 (7.48,32.07)	34.55 (8.77,43.26)	20.34 (4.95,26.74)	<0.0001

Further regional analysis revealed statistically significant differences in the median ASMR across geographic regions (Kruskal-Wallis test, *p* < 0.001). The burden of mortality showed substantial variation: the median ASMR in Europe (27.36/100,000) and North America (28.45/100,000) was approximately three times higher than that in Latin America and the Caribbean (9.53/100,000). This pronounced geographic disparity, coupled with the observed income correlation, suggests a significant association between socioeconomic and geographic factors influencing the disease burden, highlighting substantial regional health inequalities.

### 3.5 Sex differences in respiratory tumor mortality rates across regions

**Figure 5:**
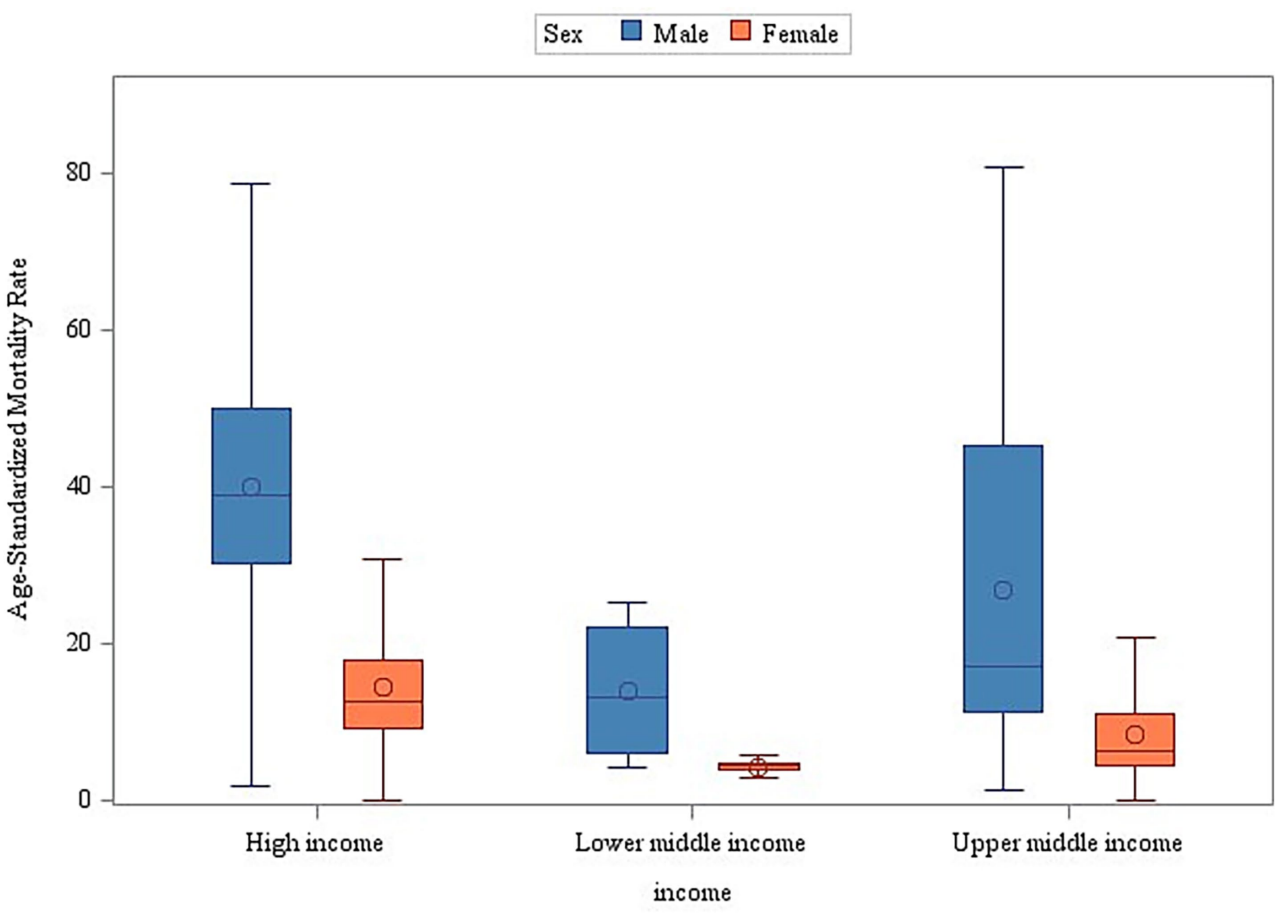
Sex and region as determinants of age-standardized mortality disparities (2000–2019).

**Figure 6:**
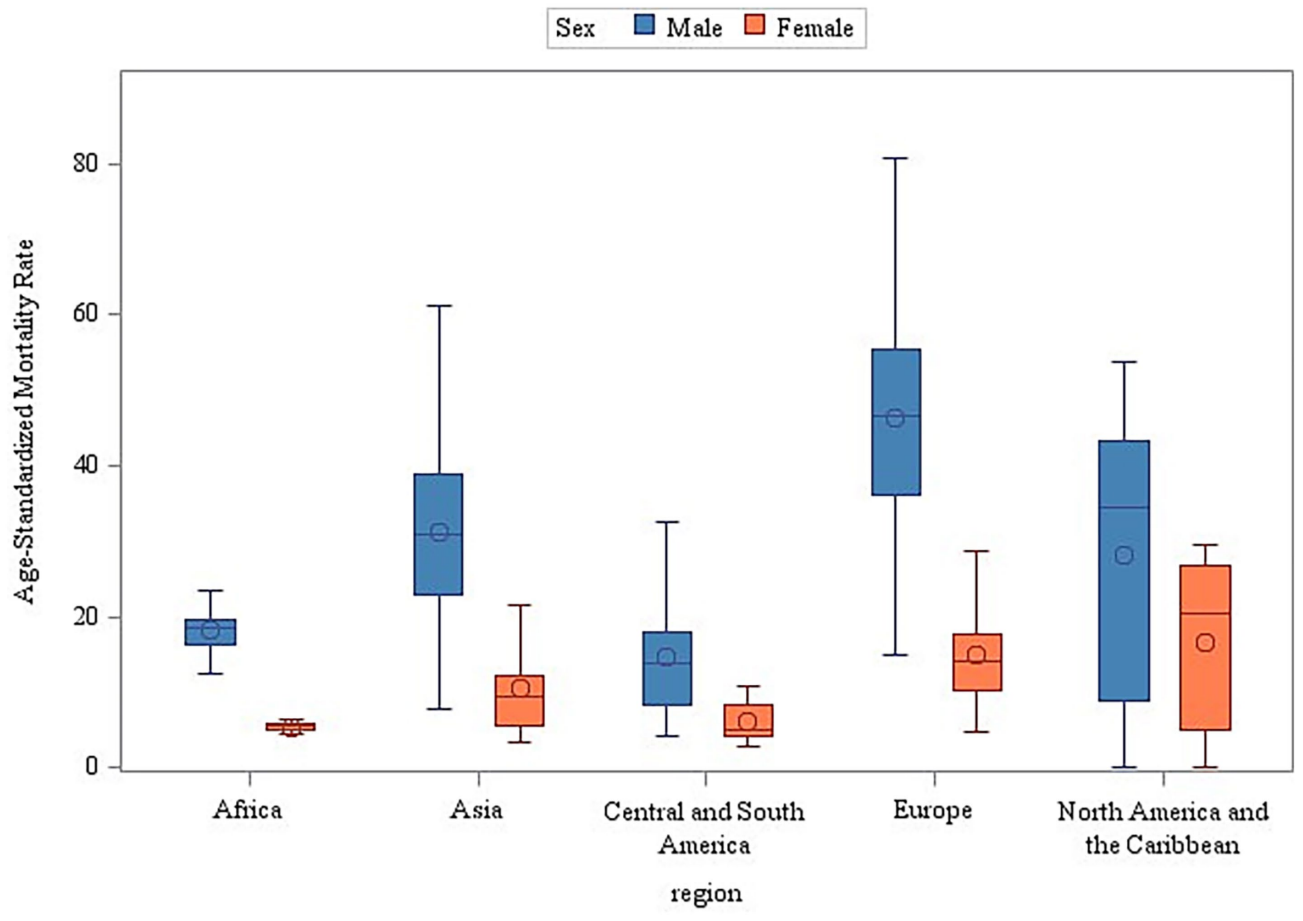
Sex and income stratification of age-adjusted mortality: a global perspective (2000–2019).

The stratified analyses across geographic regions and income levels consistently demonstrated significantly higher age-standardized mortality rates (ASMR) for male individuals compared to female individuals (Wilcoxon rank-sum test, *p* < 0.001) (Figures 5, 6). Among the 50 analyzed countries, Iceland was the only exception without a statistically significant sex difference (Male ASMR: 29.03/100,000 vs. Female ASMR: 27.79/100,000; *p* = 0.172). In contrast, the remaining 49 countries exhibited significantly higher male mortality rates (*p* < 0.05) (see Supplementary Table 3 and Supplementary Figure 2 for detailed country-level data and visualizations).

## 4 Discussion

Respiratory cancers represent a major global public health challenge. Their high incidence, mortality, and associated socioeconomic burden establish them as a central focus for cancer prevention and control efforts globally (9). Among malignant tumors, lung cancer consistently ranks first in the global disease burden (10). Our analysis revealed a significant downward trend in the global average age-standardized mortality rate (ASMR) for respiratory cancers between 2000 and 2019, decreasing from 24.0 to 19.0 per 100,000 population. This finding aligns with the declining global trend reported by Ma et al. (11), who documented a decrease in the ASMR from 27.8 per 100,000 population in 1990 to 22.8 per 100,000 population in 2019. This sustained reduction likely reflects the impact of global tobacco control initiatives, notably the WHO Framework Convention on Tobacco Control (FCTC) (12), alongside significant therapeutic advances for these malignancies. Despite this encouraging decline, the persistently high absolute burden of respiratory cancers remains a significant public health concern.

Comparative regional analysis revealed a pronounced disparity in respiratory cancer mortality burdens, with median mortality rates in Europe and North America approximately three times higher than in Latin America and the Caribbean (*p* < 0.001). This significant inequality reflects the complex interplay of multiple determinants: Environmental and Behavioral Factors: Industrial pollution in Europe and North America (13) and dietary patterns (e.g., high-calorie diets) in North America (14); Healthcare System Variations: Higher rates of early detection through screening programs in high-income regions, contrasted with potential underreporting and diagnostic limitations in resource-constrained settings; and Socioeconomic Contexts: Aging populations in high-income regions versus competing health priorities and weaker infectious disease control in parts of Latin America and the Caribbean (15). Furthermore, the composition of the Global Respiratory Tumor Mortality Registry System (GRTMRS) itself reflects a bias, being predominantly comprised of high-income (68%) and European (52%) countries. This over-representation likely masks the true burden in many lower-income regions, where suboptimal diagnostic capabilities lead to significant underdetection of lung cancer (16) and respiratory cancers often rank lower among public health priorities (17). Less robust health systems, limited treatment options, and inadequate infrastructure hinder effective management (1). These findings underscore the necessity for regionally tailored interventions, such as strengthening primary prevention (e.g., tobacco taxation, dietary interventions) targeting chronic disease risk factors in Europe and North America and enhancing primary care surveillance systems to reduce underdiagnosis and mitigate delays in diagnosis and treatment initiation in Latin America and the Caribbean. Implementing such differentiated strategies is critical for systematically addressing these profound regional health inequities. The present study found that income class was significantly positively associated with respiratory tumor mortality, challenging the traditional theory of “higher income, better health.” This suggests that the higher the regional income level, the higher the standardized mortality rate (ASMR) of respiratory tumors. Potential mechanisms include lagged effects of historical tobacco and occupational exposure in high-income areas (e.g., mid-20th century smoking peaks in Europe and the United States (18)), diagnostic bias due to widespread medical screening (e.g., overdiagnosis by low-dose CT (19)), and potential “reverse causation” (disease-induced poverty) in cross-sectional data.

To address these disparities, we propose regionally tailored strategies. For high-income countries (Europe/USA), it is necessary to strengthen primary prevention by implementing tobacco taxation and dietary interventions targeting chronic disease risk factors. In addition, optimizing treatment pathways through the development of evidence-based criteria for “clinically significant lung cancer” (e.g., incorporating tumor volume doubling time) can help mitigate over-treatment. For LMICs, it is necessary to enhance early detection by strengthening primary healthcare surveillance systems to reduce under-diagnosis and diagnostic/treatment delays; implement resource-adapted screening models, such as stepwise screening models (e.g., Cameroon’s sputum cytology → CT referral cascade); leverage fiscal policies, including scale mechanisms such as Brazil’s “tobacco tax-healthcare transfer,” which allocates 30% of revenue to primary care screening; and facilitate access to diagnostic by promote diagnostic technology gradient transfer (e.g., Indonesia’s 60% cost reduction via pre-owned CT imports) (20). These coordinated interventions leverage economic mechanisms to optimize resource allocation and systematically reduce regional health inequities.

Our stratified analyses consistently demonstrated significantly higher age-standardized mortality rates (ASMR) for respiratory cancers among male individuals versus female individuals across geographic regions and income levels (*p* < 0.001). This persistent sex-independent mortality gradient suggests that fundamental biological and behavioral distinctions transcend socioeconomic contexts. The disparity aligns with the Global Burden of Disease (GBD) 2019 data attributing 76.2% of male lung cancer deaths to smoking compared to 38.9% in female individuals, indicating that smoking accounts for approximately twice the mortality burden in men. Country-specific studies (e.g., Nepal) corroborate tobacco as the primary driver of elevated male ASMR (6). Biological differences may contribute to this gap: although some evidence suggests enhanced anti-tumor immunity in male individuals, this potential advantage is likely negated by higher exposure to carcinogens (tobacco, occupational hazards) (21). Conversely, female individuals demonstrate greater health awareness and healthcare engagement, leading to earlier diagnoses (e.g., a 15% higher Stage I detection rate). Symptom minimization among male individuals frequently delays treatment initiation (22). Notably, rising female incidence in low-income regions highlights emerging risks. Secondhand smoke exposure represents a significant etiology for non-smoking female individuals, underscoring how sex-specific risk profiles evolve with societal changes (23). Therefore, respiratory cancer mortality disparities arise from a tripartite interaction of biological factors (e.g., hormonal pathways and immune responses), behavioral patterns (e.g., smoking habits and healthcare-seeking behaviors), and healthcare accessibility (e.g., early detection and treatment responsiveness). Effective prevention requires transcending unisex policy frameworks through biologically informed interventions, such as targeted risk mitigation—exemplified by Chile’s silicosis surtax, which penalizes mining companies based on workforce lung cancer incidence—and exposure reduction, as seen in India’s clean stove initiatives, which decreased female lung cancer risk by 40% through reduced indoor air pollution. Screening protocols must move beyond smoking-centric paradigms. For example, Taiwan (China) incorporates a * ≥ 20-year cooking history* into its female screening criteria (24), while European guidelines mandate universal EGFR mutation testing for non-smoking women. Integrating molecular insights into public health practice transforms biological distinctions from barriers to health equity into opportunities for precision medicine—ensuring vulnerability-driven, rather than sex-blinded, interventions.

The cornerstone of global tobacco control remains the WHO Framework Convention on Tobacco Control (FCTC), ratified by the World Health Assembly in 2003 and enacted in 2005 (25). Its evidence-based MPOWER strategy provides the operational framework for tobacco control: Monitor tobacco use and prevention policies, protect people from tobacco smoke, offer help to quit tobacco use, warn about the dangers of tobacco, enforce bans on advertising, promotion, and sponsorship, and raise taxes on tobacco. According to the WHO Report on the Global Tobacco Epidemic 2025 (26), MPOWER measures now cover over 5.6 billion people. Nevertheless, sustained implementation remains critical for continued progress. High-income countries (HICs) require targeted interventions despite having established control systems. These interventions should focus on addressing persistently elevated smoking prevalence among adult male individuals and vulnerable subgroups, as well as counteracting declining rates of smoking reduction through enhanced economic disincentives, integration with mental health and social services, and regulatory innovations disrupting smoking’s cultural normalization. Low- and middle-income countries (LMICs) should prioritize developing cost-effective tobacco surveillance systems and building capacity for the early diagnosis of tobacco-related diseases (notably cancer and COPD) through standardized population surveys, community-level screening programs, and tiered care models with low-cost diagnostic technologies. These context-specific recommendations translate epidemiological insights into actionable strategies for reducing the global burden of tobacco-related diseases.

This study has several limitations. First, we did not adjust for key confounders including smoking prevalence, ambient air pollution (e.g., PM_₂.₅_), and occupational carcinogen exposures. Future longitudinal cohort studies should establish temporal relationships between socioeconomic transitions and lung cancer risk while optimizing two critical public health interventions: Risk-stratified screening for precise high-risk population identification and environmental governance through targeted PM_₂.₅_ control measures. Such approaches could disrupt the socioenvironmental risk cascade linking economic development to environmental hazards.

In addition, prospective cohorts should elucidate causal pathways underlying sex-specific mortality disparities to enable evidence-based interventions reducing health inequities. In summary, respiratory tumor mortality in high- and middle-income countries globally from 2000 to 2019 was likely positively associated with income level, and male individuals were likely to have higher standardized mortality rates than female individuals. These findings highlight the urgent need for targeted prevention strategies and equitable global surveillance systems to address persistent disparities in respiratory cancer outcomes.

## Data Availability

The original contributions presented in the study are included in the article/Supplementary material, further inquiries can be directed to the corresponding author.

## Footnotes

^1^https://platform.who.int/mortality, accessed 4 March 2025.
