# Supplementary material for: Global respiratory tumor mortality correlation study with economic level, 2000–2019
Source: Front Public Health. 2025 Aug 19;13:1647634. doi: 10.3389/fpubh.2025.1647634 (PMC12402828; doi:10.3389/fpubh.2025.1647634)
Supplement: Supplementary file 1 [file Data_Sheet_1.docx]

Table 1**:** Unavailable data (🗶) for Trachea, bronchus, lung cancers by country and year（n=81）

| **Country Name** | **2000** | **2001** | **2002** | **2003** | **2004** | **2005** | **2006** | **2007** | **2008** | **2009** | **2010** | **2011** | **2012** | **2013** | **2014** | **2015** | **2016** | **2017** | **2018** | **2019** | **入组连续性(%)** | **数据可用性（%）** |
| --- | --- | --- | --- | --- | --- | --- | --- | --- | --- | --- | --- | --- | --- | --- | --- | --- | --- | --- | --- | --- | --- | --- |
| **Antigua and Barbuda** |  |  |  |  |  |  |  |  |  |  |  |  |  |  |  |  |  |  |  |  | **100.0** | **79.0** |
| **Argentina** |  |  |  |  |  |  |  |  |  |  |  |  |  |  |  |  |  |  |  |  | **100.0** | **72.4** |
| **Armenia** |  |  |  |  | **×** | **×** |  |  |  |  |  |  |  |  |  |  |  |  |  |  | **90.0** | **92.2** |
| **Australia** |  |  |  |  |  | **×** |  |  |  |  |  |  |  |  |  |  |  |  |  |  | **95.0** | **91.6** |
| **Austria** |  |  |  |  |  |  |  |  |  |  |  |  |  |  |  |  |  |  |  |  | **100.0** | **87.5** |
| **Bahamas** |  |  |  |  |  |  |  |  |  |  |  |  |  |  |  |  | **×** | **×** | **×** | **×** | **80.0** | **76.7** |
| **Belarus** |  |  |  |  | **×** | **×** | **×** |  |  |  |  |  | **×** |  |  | **×** | **×** | **×** |  | **×** | **60.0** | **90.1** |
| **Belgium** |  |  |  |  |  |  |  |  |  |  |  |  |  |  |  |  |  |  |  |  | **100.0** | **80.6** |
| **Belize** |  |  |  |  |  |  |  |  |  |  |  |  |  |  |  |  |  | **×** | **×** | **×** | **85.0** | **89.3** |
| **Bosnia and Herzegovina** | **×** | **×** | **×** | **×** | **×** | **×** | **×** | **×** | **×** | **×** | **×** |  | **×** | **×** |  | **×** |  |  |  |  | **30.0** | **71.8** |
| **Brazil** |  |  |  |  |  |  |  |  |  |  |  |  |  |  |  |  |  |  |  |  | **100.0** | **84.3** |
| **Brunei Darussalam** |  |  |  |  |  |  |  |  |  |  |  |  |  |  |  |  |  |  |  |  | **100.0** | **71.0** |
| **Canada** |  |  |  |  |  |  |  |  |  |  |  |  |  |  |  |  |  |  |  |  | **100.0** | **91.6** |
| **Chile** |  |  |  |  |  |  |  |  |  |  |  |  |  |  |  |  |  |  |  |  | **100.0** | **89.2** |
| **Colombia** |  |  |  |  |  |  |  |  |  |  |  |  |  |  |  |  |  |  |  |  | **100.0** | **91.4** |
| **Costa Rica** |  |  |  |  |  |  |  |  |  |  |  |  |  |  |  |  |  |  |  |  | **100.0** | **89.4** |
| **Croatia** |  |  |  |  |  |  |  |  |  |  |  |  |  |  |  |  |  |  |  |  | **100.0** | **90.1** |
| **Cuba** |  |  |  |  |  |  |  |  |  |  |  |  |  |  |  |  |  |  |  |  | **100.0** | **92.4** |
| **Cyprus** |  | **×** | **×** | **×** |  |  |  |  |  |  |  |  |  |  |  |  |  |  |  |  | **85.0** | **82.8** |
| **Czechia** |  |  |  |  |  |  |  |  |  |  |  |  |  |  |  |  |  |  |  |  | **100.0** | **86.2** |
| **Denmark** |  |  |  |  |  |  |  |  |  |  |  |  |  |  |  |  |  |  |  |  | **100.0** | **84.0** |
| **Dominica** |  |  |  |  |  |  |  |  |  |  |  |  |  |  |  |  |  |  |  |  | **100.0** | **70.5** |
| **Ecuador** |  |  |  |  |  |  |  |  |  |  |  |  |  |  |  |  |  |  |  |  | **100.0** | **73.4** |
| **Estonia** |  |  |  |  |  |  |  |  |  |  |  |  |  |  |  |  |  |  |  |  | **100.0** | **92.9** |
| **Finland** |  |  |  |  |  |  |  |  |  |  |  |  |  |  |  |  |  |  |  |  | **100.0** | **97.4** |
| **France** |  |  |  |  |  |  |  |  |  |  |  |  |  |  |  |  |  |  |  |  | **100.0** | **79.8** |
| **Georgia** |  |  | **×** | **×** |  |  |  |  | **×** |  |  |  |  |  |  |  |  |  |  |  | **85.0** | **64.3** |
| **Germany** |  |  |  |  |  |  |  |  |  |  |  |  |  |  |  |  |  |  |  |  | **100.0** | **85.9** |
| **Greece** |  |  |  |  |  |  |  |  |  |  |  |  |  |  |  |  |  |  |  |  | **100.0** | **75.0** |
| **Grenada** | **×** |  |  |  |  |  |  |  |  |  |  |  |  |  |  |  |  |  |  |  | **95.0** | **89.2** |
| **Guatemala** |  |  |  |  |  |  |  |  |  |  |  |  |  |  |  |  |  |  |  |  | **100.0** | **68.5** |
| **Guyana** | **×** |  |  |  |  |  |  |  |  |  |  |  |  |  |  |  |  |  |  |  | **95.0** | **69.8** |
| **Hungary** |  |  |  |  |  |  |  |  |  |  |  |  |  |  |  |  |  |  |  |  | **100.0** | **92.9** |
| **Iceland** |  |  |  |  |  |  |  |  |  |  |  |  |  |  |  |  |  |  |  |  | **100.0** | **91.5** |
| **Iran (Islamic Republic of)** | **×** | **×** | **×** | **×** | **×** | **×** | **×** | **×** | **×** | **×** | **×** | **×** | **×** |  |  |  |  |  | **×** | **×** | **25.0** | **72.9** |
| **Ireland** |  |  |  |  |  |  |  |  |  |  |  |  |  |  |  |  |  |  |  |  | **100.0** | **93.0** |
| **Israel** |  |  |  |  |  |  |  |  |  |  |  |  |  |  |  |  |  |  |  |  | **100.0** | **79.1** |
| **Italy** |  |  |  |  |  |  |  |  |  |  |  |  |  |  |  |  |  |  |  |  | **100.0** | **88.0** |
| **Jamaica** |  |  |  |  |  |  |  | **×** | **×** |  |  |  |  |  |  | **×** | **×** | **×** | **×** | **×** | **65.0** | **90.0** |
| **Japan** |  |  |  |  |  |  |  |  |  |  |  |  |  |  |  |  |  |  |  |  | **100.0** | **79.0** |
| **Jordan** | **×** | **×** | **×** | **×** | **×** | **×** | **×** | **×** |  |  |  |  |  | **×** | **×** |  |  |  |  | **×** | **45.0** | **68.0** |
| **Kazakhstan** |  |  |  |  |  |  |  |  |  |  |  |  |  |  |  |  |  |  |  |  | **100.0** | **89.0** |
| **Kuwait** |  |  |  |  |  |  |  |  |  |  |  |  |  |  |  |  |  |  |  |  | **100.0** | **85.0** |
| **Kyrgyzstan** |  |  |  |  |  |  |  |  |  |  |  |  |  |  |  |  |  |  |  |  | **100.0** | **94.2** |
| **Latvia** |  |  |  |  |  |  |  |  |  |  |  |  |  |  |  |  |  |  |  |  | **100.0** | **94.7** |
| **Lithuania** |  |  |  |  |  |  |  |  |  |  |  |  |  |  |  |  |  |  |  |  | **100.0** | **95.1** |
| **Luxembourg** |  |  |  |  |  |  |  |  |  |  |  |  |  |  |  |  |  |  |  |  | **100.0** | **83.6** |
| **Malta** |  |  |  |  |  |  |  |  |  |  |  |  |  |  |  |  |  |  |  |  | **100.0** | **92.4** |
| **Mauritius** |  |  |  |  |  |  |  |  |  |  |  |  |  |  |  |  |  |  |  |  | **100.0** | **89.5** |
| **Mexico** |  |  |  |  |  |  |  |  |  |  |  |  |  |  |  |  |  |  |  |  | **100.0** | **91.6** |
| **Mongolia** | **×** | **×** | **×** | **×** | **×** | **×** | **×** | **×** | **×** | **×** | **×** | **×** | **×** | **×** | **×** | **×** |  |  |  |  | **20.0** | **81.2** |
| **Netherlands** |  |  |  |  |  |  |  |  |  |  |  |  |  |  |  |  |  |  |  |  | **100.0** | **84.0** |
| **New Zealand** |  |  |  |  |  |  |  |  |  |  |  |  |  |  |  |  |  | **×** | **×** | **×** | **85.0** | **95.5** |
| **Nicaragua** | **×** | **×** | **×** | **×** | **×** |  |  |  |  |  |  |  |  |  |  |  |  |  |  |  | **75.0** | **86.8** |
| **Norway** |  |  |  |  |  |  |  |  |  |  |  |  |  |  |  |  |  | **×** | **×** | **×** | **85.0** | **86.7** |
| **Panama** |  |  |  |  |  |  |  |  |  |  |  |  |  |  |  |  |  |  |  |  | **100.0** | **81.3** |
| **Paraguay** |  |  |  |  |  |  |  |  |  |  |  |  |  |  |  |  |  |  |  |  | **100.0** | **81.8** |
| **Philippines** |  |  |  |  | **×** | **×** |  |  |  |  |  |  | **×** | **×** |  | **×** |  |  |  |  | **75.0** | **83.7** |
| **Poland** |  |  |  |  |  |  |  |  |  |  |  |  |  |  |  |  |  |  |  |  | **100.0** | **70.7** |
| **Portugal** |  |  |  |  | **×** | **×** | **×** |  |  |  |  |  |  |  |  |  |  |  |  |  | **85.0** | **82.7** |
| **Republic of Korea** |  |  |  |  |  |  |  |  |  |  |  |  |  |  |  |  |  |  |  |  | **100.0** | **81.0** |
| **Republic of Moldova** |  |  |  |  |  |  |  |  |  |  |  |  |  |  |  |  |  |  |  | **×** | **95.0** | **97.9** |
| **Romania** |  |  |  |  |  |  |  |  |  |  |  |  |  |  |  |  |  |  |  |  | **100.0** | **81.7** |
| **Saint Kitts and Nevis** |  |  |  |  |  |  |  |  |  |  |  |  |  |  |  |  |  | **×** | **×** | **×** | **85.0** | **78.9** |
| **Saint Lucia** |  |  |  |  |  |  |  | **×** |  |  |  |  |  |  |  |  |  |  |  |  | **95.0** | **86.0** |
| **Saint Vincent and the Grenadines** |  |  |  |  |  |  |  |  |  |  |  |  |  |  |  |  |  |  |  |  | **100.0** | **85.1** |
| **Serbia** |  |  |  |  |  |  |  |  |  |  |  |  |  |  |  |  |  |  |  |  | **100.0** | **79.7** |
| **Singapore** |  |  |  |  |  |  |  |  |  |  |  |  |  |  |  |  |  |  |  |  | **100.0** | **95.1** |
| **Slovakia** |  |  |  |  |  |  |  |  |  |  |  | **×** |  |  |  | **×** |  |  |  |  | **90.0** | **91.4** |
| **Slovenia** |  |  |  |  |  |  |  |  |  |  |  |  |  |  |  |  |  |  |  |  | **100.0** | **86.7** |
| **South Africa** |  |  |  |  |  |  |  |  |  |  |  |  |  |  |  |  |  |  |  | **×** | **95.0** | **73.3** |
| **Spain** |  |  |  |  |  |  |  |  |  |  |  |  |  |  |  |  |  |  |  |  | **100.0** | **86.8** |
| **Suriname** |  |  |  |  |  |  |  |  |  |  |  |  |  |  |  | **×** | **×** | **×** | **×** | **×** | **75.0** | **69.9** |
| **Sweden** |  |  |  |  |  |  |  |  |  |  |  |  |  |  |  |  |  |  |  |  | **100.0** | **86.9** |
| **Switzerland** |  |  |  |  |  |  |  |  |  |  |  |  |  |  |  |  |  |  |  |  | **100.0** | **86.8** |
| **Tajikistan** |  |  |  |  |  |  | **×** | **×** | **×** | **×** | **×** | **×** | **×** | **×** | **×** | **×** |  |  | **×** | **×** | **40.0** | **69.1** |
| **Thailand** |  | **×** |  |  |  |  |  |  |  |  |  |  |  |  |  |  |  |  |  |  | **95.0** | **61.5** |
| **United States of America** |  |  |  |  |  |  |  |  |  |  |  |  |  |  |  |  |  |  |  |  | **100.0** | **87.9** |
| **Uruguay** |  |  |  |  |  |  |  |  |  |  |  | **×** |  |  |  |  |  |  |  |  | **95.0** | **79.7** |
| **Uzbekistan** |  |  |  |  |  |  | **×** | **×** | **×** |  |  |  |  |  |  |  |  |  |  |  | **85.0** | **83.6** |
| **Venezuela (Bolivarian Republic of)** |  |  |  |  |  |  |  |  |  |  |  |  |  |  |  |  |  | **×** | **×** | **×** | **85.0** | **88.7** |

Table1**:** Unavailable data (🗶) for Trachea, bronchus, lung cancers by country and year

Table 2. Geographical Distribution and Income Level Classification of Included Countries（n=50）

| Country Name | Geographical distribution | income |
| --- | --- | --- |
| Mauritius | Africa | Upper middle income |
| Brunei Darussalam | Asia | High income |
| Israel | Asia | High income |
| Japan | Asia | High income |
| Kuwait | Asia | High income |
| Republic of Korea | Asia | High income |
| Singapore | Asia | High income |
| Kyrgyzstan | Asia | Lower middle income |
| Kazakhstan | Asia | Upper middle income |
| Argentina | Central and South America | High income |
| Chile | Central and South America | High income |
| Guatemala | Central and South America | Lower middle income |
| Brazil | Central and South America | Upper middle income |
| Colombia | Central and South America | Upper middle income |
| Costa Rica | Central and South America | Upper middle income |
| Ecuador | Central and South America | Upper middle income |
| Mexico | Central and South America | Upper middle income |
| Panama | Central and South America | Upper middle income |
| Paraguay | Central and South America | Upper middle income |
| Austria | Europe | High income |
| Belgium | Europe | High income |
| Czechia | Europe | High income |
| Denmark | Europe | High income |
| Estonia | Europe | High income |
| Finland | Europe | High income |
| France | Europe | High income |
| Germany | Europe | High income |
| Greece | Europe | High income |
| Hungary | Europe | High income |
| Iceland | Europe | High income |
| Ireland | Europe | High income |
| Italy | Europe | High income |
| Latvia | Europe | High income |
| Lithuania | Europe | High income |
| Luxembourg | Europe | High income |
| Malta | Europe | High income |
| Netherlands | Europe | High income |
| Poland | Europe | High income |
| Slovenia | Europe | High income |
| Spain | Europe | High income |
| Sweden | Europe | High income |
| Switzerland | Europe | High income |
| Croatia | Europe | Upper middle income |
| Romania | Europe | Upper middle income |
| Serbia | Europe | Upper middle income |
| Antigua and Barbuda | North America and the Caribbean | High income |
| Canada | North America and the Caribbean | High income |
| United States of America | North America and the Caribbean | High income |
| Cuba | North America and the Caribbean | Upper middle income |
| Saint Vincent and the Grenadines | North America and the Caribbean | Upper middle income |

Figure 1: Mortality rates in various countries in different years

**Table 3. Cross-National Comparison of Sex Differences in Tracheal/Bronchial/Lung Cancer Case Fatality Rates (2000–2019, Wilcoxon Rank-Sum Test)**

| country | Geographical distribution | income | MQ_all | MQ_male | MQ_female | p |
| --- | --- | --- | --- | --- | --- | --- |
| Hungary | Europe | High income | 49.52(47.24,50.29) | 78.65(71.25,83.18) | 29.24(25.03,30.79) | <.0001 |
| Serbia | Europe | Upper middle income | 37.67(35.34,38.27) | 60.17(57.24,62.08) | 18.65(14.67,19.7) | <.0001 |
| Poland | Europe | High income | 37.13(34.98,39.06) | 64.57(56.68,72.57) | 17.74(15.58,19.48) | <.0001 |
| Denmark | Europe | High income | 35.37(32.25,38.32) | 40.61(36.09,46.72) | 30.78(29.32,32.31) | <.0001 |
| Croatia | Europe | Upper middle income | 34.66(33.84,35.11) | 61.77(57.2,65.21) | 14.02(12.72,15.66) | <.0001 |
| Netherlands | Europe | High income | 34.65(31.42,35.42) | 48.38(39.7,54.02) | 23.98(22.15,25.24) | <.0001 |
| Canada | North America and the Caribbean | High income | 33.73(30.14,36.27) | 40.55(34.66,46.14) | 27.63(26.53,28.55) | <.0001 |
| United States of America | North America and the Caribbean | High income | 33.33(28.64,37.35) | 41.45(34.55,48.31) | 26.87(23.83,28.86) | <.0001 |
| Belgium | Europe | High income | 32.45(29.23,33.49) | 53.84(44.14,58.73) | 15.56(13.66,16.73) | <.0001 |
| Cuba | North America and the Caribbean | Upper middle income | 30.2(29.39,31.26) | 41.28(40.62,44) | 20.38(19.57,20.68) | <.0001 |
| Czechia | Europe | High income | 29.97(25.69,33.76) | 50.29(40.03,59.85) | 14.63(14.33,15.02) | <.0001 |
| Slovenia | Europe | High income | 29.73(28.74,31.21) | 48.81(43.39,56.07) | 14.84(13.76,16.51) | <.0001 |
| Greece | Europe | High income | 29.34(28.89,29.83) | 52.81(51.4,53.79) | 9.61(8.5,11.13) | <.0001 |
| Ireland | Europe | High income | 29.07(26.58,29.42) | 37.63(32.79,40.38) | 21.3(20.64,21.84) | <.0001 |
| Iceland | Europe | High income | 28.94(25.94,29.82) | 29.03(27.31,31.08) | 27.79(25.34,29.44) | 0.1719 |
| Romania | Europe | Upper middle income | 28.58(27.66,29.14) | 51.05(50,52.23) | 9.99(9.33,11.41) | <.0001 |
| France | Europe | High income | 26.95(26.07,27.32) | 45.14(41.18,48.44) | 12.34(10.47,13.61) | <.0001 |
| Estonia | Europe | High income | 26.84(25.02,29.48) | 55.03(50.57,63.92) | 9.21(8.56,10.11) | <.0001 |
| Luxembourg | Europe | High income | 26.39(23.12,27.32) | 40.09(33.22,46.92) | 14.23(12.02,15.9) | <.0001 |
| Latvia | Europe | High income | 26.21(24.06,27.49) | 58.36(51.93,61.66) | 7.53(7.13,8.09) | <.0001 |
| Spain | Europe | High income | 25.6(24.15,26.16) | 47.04(41.83,50.55) | 7.98(6.4,9.44) | <.0001 |
| Germany | Europe | High income | 25.48(25.11,25.84) | 38.11(35.58,42.07) | 14.95(13.42,16.57) | <.0001 |
| Lithuania | Europe | High income | 25.13(23.79,26.78) | 55.03(50.24,59.09) | 6.75(6.23,7.06) | <.0001 |
| Singapore | Asia | High income | 25.11(22.25,27.18) | 37.67(33.16,42.01) | 14.58(13.1,15.62) | <.0001 |
| Italy | Europe | High income | 25.06(23.11,26.72) | 42.93(37.15,48.25) | 11.13(10.17,12.01) | <.0001 |
| Republic of Korea | Asia | High income | 24.37(22.02,27.07) | 44.2(39.44,50.02) | 11.11(10.11,11.96) | <.0001 |
| Brunei Darussalam | Asia | High income | 24.29(20.79,29.24) | 29.26(24.28,34.96) | 20.03(17.38,25.07) | 0.0020 |
| Austria | Europe | High income | 23.8(23.08,24.31) | 35.33(31.94,38.63) | 14.81(13.25,16.07) | <.0001 |
| Switzerland | Europe | High income | 21.7(20.1,22.42) | 30.72(27.47,34.85) | 14.04(12.72,14.49) | <.0001 |
| Kazakhstan | Asia | Upper middle income | 21.33(16.77,25.24) | 44.48(33.97,52.38) | 6.17(5.37,7.4) | <.0001 |
| Malta | Europe | High income | 20.38(19.62,21.62) | 37.16(32.76,39.93) | 7.67(5.79,9.13) | <.0001 |
| Japan | Asia | High income | 19.97(18.97,20.6) | 34.12(31.72,35.57) | 9.26(9.02,9.49) | <.0001 |
| Argentina | Central and South America | High income | 19.38(18.48,20.27) | 32.1(29.06,35.11) | 9.54(8.71,10.42) | <.0001 |
| Finland | Europe | High income | 19.25(18.19,20.29) | 31.34(26.79,34.86) | 10.78(9.6,11.31) | <.0001 |
| Sweden | Europe | High income | 18.7(17.53,19.28) | 21.31(18.55,23.25) | 16.64(15.78,17.2) | <.0001 |
| Israel | Asia | High income | 18.58(18.24,19.22) | 28.19(27.13,29.94) | 10.38(10.09,10.8) | <.0001 |
| Chile | Central and South America | High income | 13.65(13.1,13.89) | 19.72(18.49,21.04) | 8.92(8.49,9.48) | <.0001 |
| Brazil | Central and South America | Upper middle income | 12.66(12.38,12.75) | 18.23(17.26,18.83) | 8.49(7.58,9.31) | <.0001 |
| Kyrgyzstan | Asia | Lower middle income | 12.38(11.59,12.89) | 22.16(20.93,22.83) | 4.85(4.51,5.27) | <.0001 |
| Mauritius | Africa | Upper middle income | 10.89(10.21,11.45) | 18.46(16.29,19.69) | 5.5(5.03,5.78) | <.0001 |
| Colombia | Central and South America | Upper middle income | 10.57(9.88,11.27) | 14.21(12.88,15.41) | 7.68(7.29,7.85) | <.0001 |
| Paraguay | Central and South America | Upper middle income | 9.99(9.52,10.65) | 16.56(15.89,17.65) | 3.82(3.49,4.22) | <.0001 |
| Kuwait | Asia | High income | 8.41(7.27,11.06) | 11.92(10.27,13.42) | 4.79(4.07,5.8) | <.0001 |
| Panama | Central and South America | Upper middle income | 8.14(7.6,9.48) | 11.63(10.51,13.56) | 5.14(4.25,5.73) | <.0001 |
| Mexico | Central and South America | Upper middle income | 7.53(6.49,9.38) | 10.89(9.03,14.07) | 4.75(4.39,5.47) | <.0001 |
| Saint Vincent and the Grenadines | North America and the Caribbean | Upper middle income | 7.19(5.18,9.98) | 8.35(6.93,13.53) | 5.25(3.47,7.08) | 0.0036 |
| Costa Rica | Central and South America | Upper middle income | 6.23(5.66,7.34) | 9.07(7.89,10.59) | 4.05(3.53,4.64) | <.0001 |
| Ecuador | Central and South America | Upper middle income | 5.83(5.43,6.13) | 7.62(6.36,7.87) | 4.5(4.1,4.72) | <.0001 |
| Antigua and Barbuda | North America and the Caribbean | High income | 5.29(3.16,7.63) | 7.89(3.97,10.49) | 2.96(2.21,4.7) | 0.0043 |
| Guatemala | Central and South America | Lower middle income | 4.85(3.95,5.55) | 6(4.77,7) | 3.9(3.26,4.37) | <.0001 |


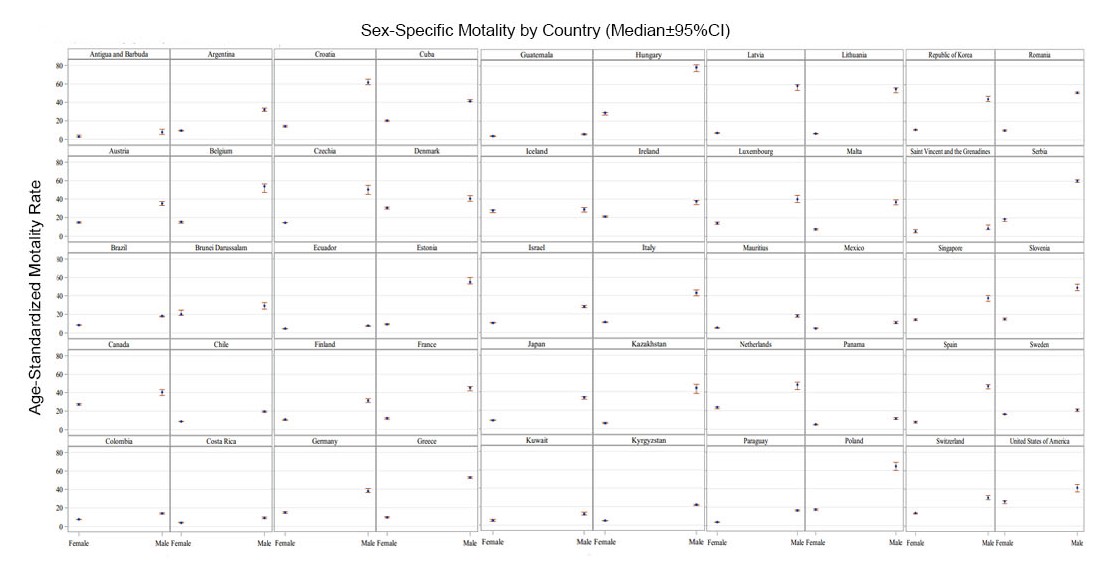
Figure 2 Intersection of Sex and Nationality in Mortality Disparities: Age-Standardized Rates (2000–2019)
